# Prevalence and associated factors of tuberculosis and diabetes mellitus comorbidity: A systematic review

**DOI:** 10.1371/journal.pone.0175925

**Published:** 2017-04-21

**Authors:** Mahteme Haile Workneh, Gunnar Aksel Bjune, Solomon Abebe Yimer

**Affiliations:** 1 Institute of Health and Society, Faculty of Medicine, University of Oslo, Oslo, Norway; 2 Amhara Regional State Health Bureau, Bahir-Dar, Ethiopia; 3 Department of Microbiology, Oslo University Hospital, Oslo, Norway; 4 Department of Bacteriology and Immunology, Norwegian Institute of Public Health, Oslo, Norway; University of Cape Town, SOUTH AFRICA

## Abstract

**Introduction:**

The dual burden of tuberculosis (TB) and diabetes mellitus (DM) has become a major global public health concern. There is mounting evidence from different countries on the burden of TB and DM comorbidity. The objective of this systematic review was to summarize the existing evidence on prevalence and associated/risk factors of TBDM comorbidity at global and regional levels.

**Methods:**

Ovid Medline, Embase, Global health, Cochrane library, Web of science and Scopus Elsevier databases were searched to identify eligible articles for the systematic review. Data were extracted using standardized excel form and pilot tested. Median with interquartile range (IQR) was used to estimate prevalence of TBDM comorbidity. Associated/risk factors that were identified from individual studies were thematically analyzed and described.

**Results:**

The prevalence of DM among TB patients ranged from 1.9% to 45%. The overall median global prevalence was 16% (IQR 9.0%-25.3%) Similarly, the prevalence of TB among DM patients ranged from 0.38% to 14% and the overall median global prevalence was 4.1% (IQR 1.8%-6.2%). The highest prevalence of DM among TB patients is observed in the studied countries of Asia, North America and Oceania. On the contrary, the prevalence of TB among DM patients is low globally, but relatively higher in the studied countries of Asia and the African continents. Sex, older age, urban residence, tobacco smoking, sedentary lifestyle, poor glycemic control, having family history of DM and TB illness were among the variables identified as associated/risk factors for TBDM comorbidity.

**Conclusion:**

This systematic review revealed that there is a high burden of DM among TB patients at global level. On the contrary, the global prevalence of TB among DM patients is low. Assessing the magnitude and risk/associated factors of TBDM comorbidity at country/local level is crucial before making decisions to undertake TBDM integrated services.

## Introduction

Diabetes mellitus (DM) and tuberculosis (TB) are major killers of mankind across the globe [1]. The World Health Organization (WHO) global report for 2015 indicates that, there were 10.4 million new cases and 1.4 million deaths resulting from TB [2]. In the same year, 415 million cases and 5.0 million deaths due to DM were registered [3]. About 95% of TB and 75% of the DM cases live in low- and middle income countries. The rising prevalence of DM is a potential threat to TB control. Poorly controlled DM increases the risk of TB and leads to unfavorable TB treatment outcomes [4–5].

WHO has recommended a collaborative framework for the clinical management and control of TBDM comorbidity. Three important intervention strategies namely, establishing mechanisms of collaboration between TB and DM control programs, detection and management of TB in patients with DM, and detection and management of DM in TB patients have been recommended [6]. Some countries in Asia (China and India) have piloted the TBDM collaborative framework and have demonstrated that bi-directional screening for both diseases is feasible [7–10]. It may also be important if other countries implement this strategy to reduce the dual burden of TBDM comorbidity. However, for policy making and implementation of this strategy, it is crucial to primarily understand the magnitude and associated factors of TBDM comorbidity particularly in low- and middle-income countries.

Former studies conducted in various parts of the world have shown that TBDM comorbidity has become a major public health problem. A wide range of estimates on the burden and associated factors of the two comorbid conditions and impact of DM on TB treatment outcome were reported [11–13]. For example, a previous systematic review of bidirectional screening for TBDM comorbidity reported high prevalence of DM among TB patients ranging from 1.9% to 35%. TB prevalence among DM patients ranged from 1.7% to 36% [11]. Similarly, another systematic review done on 13 observational studies reported that DM was associated with an increased risk of TB [relative risk (RR) = 3.11, 95% confidence interval (C.I.) 2.27–4.26] [12]. Several reasons motivated us to do the current systematic review. Firstly, the risk/associated factors of TBDM comorbidity were not included in the previous systematic reviews. Secondly, the numbers of individual studies on TBDM comorbidity conducted after 2010 have increased by 78% compared to the number of studies done before 2010. Thirdly, unlike the periods before six years, bi-directional screenings of TB and DM studies have been emerging from different African countries. Therefore, an updated synthesis of the prevalence and associated factors of the two comorbid conditions is important for policy making, planning and development of TBDM integrated services. This systematic review was thus conducted to summarize the existing evidence on prevalence and associated/risk factors of TBDM comorbidity.

## Methods

### Eligibility criteria

In this systematic review, we included all full text articles that involved human subjects of any age, and that determined either prevalence and risk/associated factors of DM among TB patients or prevalence and associated factors of TB among DM patients. Type of DM was not an inclusion/exclusion criteria and therefore both types were included. Studies reporting prevalence of DM among latent TB patients, prevalence of impaired glucose tolerance among TB patients and incidence of TB among DM patients were excluded from the review. In addition, pharmacological studies related to TBDM comorbid conditions, articles written other than English language, conference papers, abstracts without full texts, articles that didn’t describe journal’s name and corresponding author, articles that reported prevalence/incidence of the two comorbidity conditions stratified by socio-demographic and clinical parameters were excluded from the study.

### Search strategy and selection of studies

We searched Ovid Medline from 1946 to March 09/2016, Embase from 1947 to March 09/2016, Global health from1973 to March 09/2016, Cochrane library from 1992- March 09/2016, Web of science from 1900-March 09/2016 and Scopus elsevier from 1996-March 09/2016 using the following medical subject heading (MeSh) and text terms **(Table 1).** The full strategy was run in Endnote software. We also used hand searching to look for relevant reference lists and journals.

**Table 1:** Search strategy from the different data bases.

Data base	# Searches
Ovid MEDLINE(R) 1946 to Mar 09/2016	**1**. exp Diabetes Mellitus/ep, **2**. diabet*.tw,kf., **3**. 1 or 2, **4**.exp Tuberculosis/ep, **5**. tuberculosis. tw,kf, **6.** 4 or 5, **7**.exp Comorbidity/ or exp Prevalence/ or exp Incidence/ or exp Diagnosis / or exp Risk Factor/ or exp Epidemiology /, **8**. (co-morbid* or comorbid* or co-occurren* or prevalen* or inciden* or diagnos* or screen* or detect* or risk* or epidemiolog*). tw,kf, **9.** 7 or 8, **10**. exp Cohort Analysis/ or exp Case-Control Study/ or exp Cross-Sectional Study/ or exp Follow-Up Studies/ or exp Longitudinal Study/ or exp Retrospective Study/, **11**. (cohort stud* or case-control stud* or cross-sectional stud* or follow-up stud* or followup stud*longitudinal stud* or retrospective stud*). tw,kf, **12.** 10 or 11, **13.** 3 and 6 and 9 and 12, **14.** limit 13 to (english language and yr = "1946 -Current" Mar 09/2016)
Ovid Embase Classic+Embase 1947 to Mar 09/2016	**1.** exp Diabetes Mellitus/ep, **2**. diabet*. tw,kw., **3.** 1 or 2, **4**. exp Tuberculosis/ep, **5.** tuberculosis. tw,kw., **6.** 4 or 5, **7**. exp Comorbidity/ or exp Prevalence/ or exp Incidence/ or exp Diagnosis / or exp Risk Factor/ or exp Epidemiology /, **8**. (co-morbid* or comorbid* or co-occurren* or prevalen* or inciden* or diagnos* or screen* or detect* or risk* or epidemiolog*). tw,kw., **9.** 7 or 8, **10**. exp Cohort Analysis/ or exp Case-Control Study/ or exp Cross-Sectional Study/ or exp Follow-Up Studies/ or exp Longitudinal Study/ or exp Retrospective Study/, 1**1**. (cohort stud* or case-control stud* or cross-sectional stud* or follow-up stud* or followup stud*longitudinal stud* or retrospective stud*). tw,kw., **12.** 10 or 11, **13.** 3 and 6 and 9 and 12, **14.** limit 13 to to (english language and yr **= "**1947 -Current" Mar 09/2016)
Global health 1973 to 2016 Mar 09/2016	**1.** diabet*.mp., **2.** tuberculosis.mp., **3.** (co-morbid* or comorbid* or co-occurren* or prevalen* or inciden* or diagnos* or screen* or detect* or risk* or epidemiolog*).mp., **4.** (cohort stud* or case-control stud* or cross-sectional stud* or follow-up stud* or followup stud* or longitudinal stud* or retrospective stud*).mp., **5.** and/1-4, **6.** limit 5 to (english language and yr = "1973 -Current" Mar 09/2016)
Cochrane library 1992-present (Mar 09/2016)	**1.**MeSH descriptor: [Diabetes Mellitus] explode all trees, **2.**diabet*:ti,ab,kw (Word variations have been searched), **3.**MeSH descriptor: [Tuberculosis] explode all trees, **4.**tuberculosis:ti,ab,kw (Word variations have been searched), **5**.MeSH descriptor: [Comorbidity] explode all trees, **6**.MeSH descriptor: [Prevalence] explode all trees, **7.**MeSH descriptor: [Incidence] explode all trees, **8**.MeSH descriptor: [Diagnosis] explode all trees, **9.**MeSH descriptor: [Risk Factors] explode all trees, **10.**MeSH descriptor: [Epidemiology] explode all trees, **11.**comorbid*:ti,ab,kw or co-morbid*: ti,ab,kw or co-occurren*: ti,ab,kw or prevalen*: ti,ab,kw or inciden*: ti,ab,kw (Word variations have been searched), **12.**diagnos*: ti,ab,kw or screen*.ti,ab,kw, or detect*: ti,ab,kw (Word variations have been searched), **13**.risk*:ti,ab,kw or epidemilog*:ti,ab,kw (Word variations have been searched), **14.** ((#1 or #2) and (#3 or #4) and ({or #5-#9} or #10 or #11 or#12))
Web of science 1900-present (March 09/2016)	(TS = (diabet* AND tuberculosis AND (co-morbid* OR comorbid* OR co-occurren* OR prevalen* OR inciden* OR diagnos* OR screen* OR detect* OR risk* OR epidemiolog*) AND (cohort stud* OR case-control stud* OR cross-sectional stud* OR follow-up stud* OR followup stud* OR longitudinal stud* OR retrospective stud*))) *AND* LANGUAGE: (English) *AND* DOCUMENT TYPES: (Article OR Proceedings Paper) *Limiters*: Language: English, *Document* types: Article OR Proceedings Paper
Scopus 1996-March 09 /2016	(TITLE-ABS-KEY(diabet*) AND TITLE-ABS-KEY(tuberculosis) AND TITLE-ABS-KEY(co-morbid* OR comorbid* OR co-occurren* OR prevalen* OR inciden* OR diagnos* OR screen* OR detect* OR risk* OR epidemiolog*) AND TITLE-ABS-KEY(cohort stud* OR case-control stud* OR cross-sectional stud* OR follow-up stud* OR followup stud* OR longitudinal stud* OR retrospective stud*)) AND (LIMIT-TO(DOCTYPE,"ar") OR LIMIT-TO(DOCTYPE,"cp")) AND (LIMIT-TO(SUBJAREA,"MEDI")) AND (LIMIT-TO(LANGUAGE, "English"))

### Data extraction and risk of bias assessment

A standardized form using excel sheet was used to extract relevant information. The standardized form was pilot tested in twenty selected articles included in the study. A number of variables including study locations, years of publications, study periods, study designs, number of patients included in the study, and prevalence of TBDM and associated/risk factors were extracted from all studies included in the systematic review. The risk of bias for each study was assessed using study design, sampling technique and sample size determination methods as important domains. In addition, we considered ‘‘type of screening method used” and ‘‘time of screening” for studies that assessed prevalence of DM among TB patients. For studies that analyzed TB prevalence among DM patients, ‘‘type of TB screening method used” was considered as an important domain **(Table 2).** Some of the above domains were also used in the previously conducted systematic review (12).One reviewer (MHW) searched, extracted the data and assessed the risk of bias. Any ambiguity in the extracted and assessed information was resolved through discussion with the other author (SAY).

**Table 2:** Risk of bias assessment tools.

Variable	Methods used by the studies	Risk of bias	
		High	Low
Study design	Prospective cohort, cross-sectional or descriptive, case control, observational, population based study designs		0
	Retrospective cohort, record review and studies that did not report study design	1	
Sampling methods	Random selection		0
	Consecutive enrollment of all eligible patients & studies that did not describe sampling methods	1	
Sample size determination	Sample size determined		0
	Sample size not determined or studies that did not report how sample size was estimated	1	
Methods of DM screening among TB patients	Use of blood test alone, or use of combination methods (blood test either with urine glucose, self-report or medical record review)		0
	Studies that reported the use of self- report, urine glucose, record review methods alone or in combination, and studies that did not report methods of DM screening	1	
Timing of DM screening	Studies that screened at the time of TB diagnosis or before TB treatment was started and both before and after anti-TB treatment was started		0
	Studies that screened after TB treatment was initiated, or at the middle of TB treatment, or at the end of TB treatment period or both, and studies that did not report timing of DM screening	1	
Methods of TB screening among DM patients	Use of WHO or National TB Control Program diagnostic methods of the respective country, use of either combination or individual screening methods of either of the following methods: microbiologically determined (sputum microscopy or sputum culture), PCR, Xpert/RIF-TB test or QFT-G. Use of clinical sign and symptoms, response to treatment, chest x-ray, tuberculin skin test, histopathology in combination with one of the above mentioned diagnostic methods		0
	Studies that used ICD code, self-report, medical record review, clinical sign and symptoms, response to treatment, chest x-ray, tuberculin skin test, histopathology, broncho-alveolar lavage alone or in combination and studies that did not report methods of TB screening	1	

0 = low risk, 1 = high risk, DM = diabetes mellitus, TB = tuberculosis, WHO = World Health Organization, PCR = Polymerase chain reaction, Xpert MTB/RIF-TB = GeneXpert Rifampicin-TB, QFT-G = QuantiFERON-TB Gold, ICD = International classification of diseases

### Data analysis and syntheses

Descriptive statistics (range and median with interquartile range (IQR)) were used to summarize prevalence rates estimated from individual studies. Due to the observed wide variations in prevalence, and sample sizes used in the reviewed articles, we reported median prevalence rate based on geographical regions. The summaries were described into two groups, i.e. prevalence of DM among TB patients and prevalence of TB among DM patients. Data analyses were performed using Statistical Package for Social Science (SPSS) version 22 Armonk, New York 10504 IBM Corp. The risk/associated factors were grouped into main themes and described accordingly. In addition, findings of the studies were grouped into the different geographical regions of the world depending on where the individual studies were conducted. Each domain assessed for the risk of bias was categorized as either low or high risk of bias depending on the findings of each study. We scored 0 and 1 for low and high risk of bias, respectively. Accordingly, for studies that determined prevalence of DM among TB patients, the overall risk of study bias was calculated out of five total score points. While those with a total point of ≤ 2 were considered low risk, studies with a total value of 3–4 and 5 were considered to have moderate and high risk of bias, respectively. Similarly, for studies that analyzed prevalence of TB among DM patients, the overall risk of study bias was calculated out of four total score points. Consequently, studies that scored a total of ≤1 were considered low risk, and those with a total value of 2 and 3–4 were evaluated to have moderate and high risk of bias, respectively.

## Results

**Fig 1:**
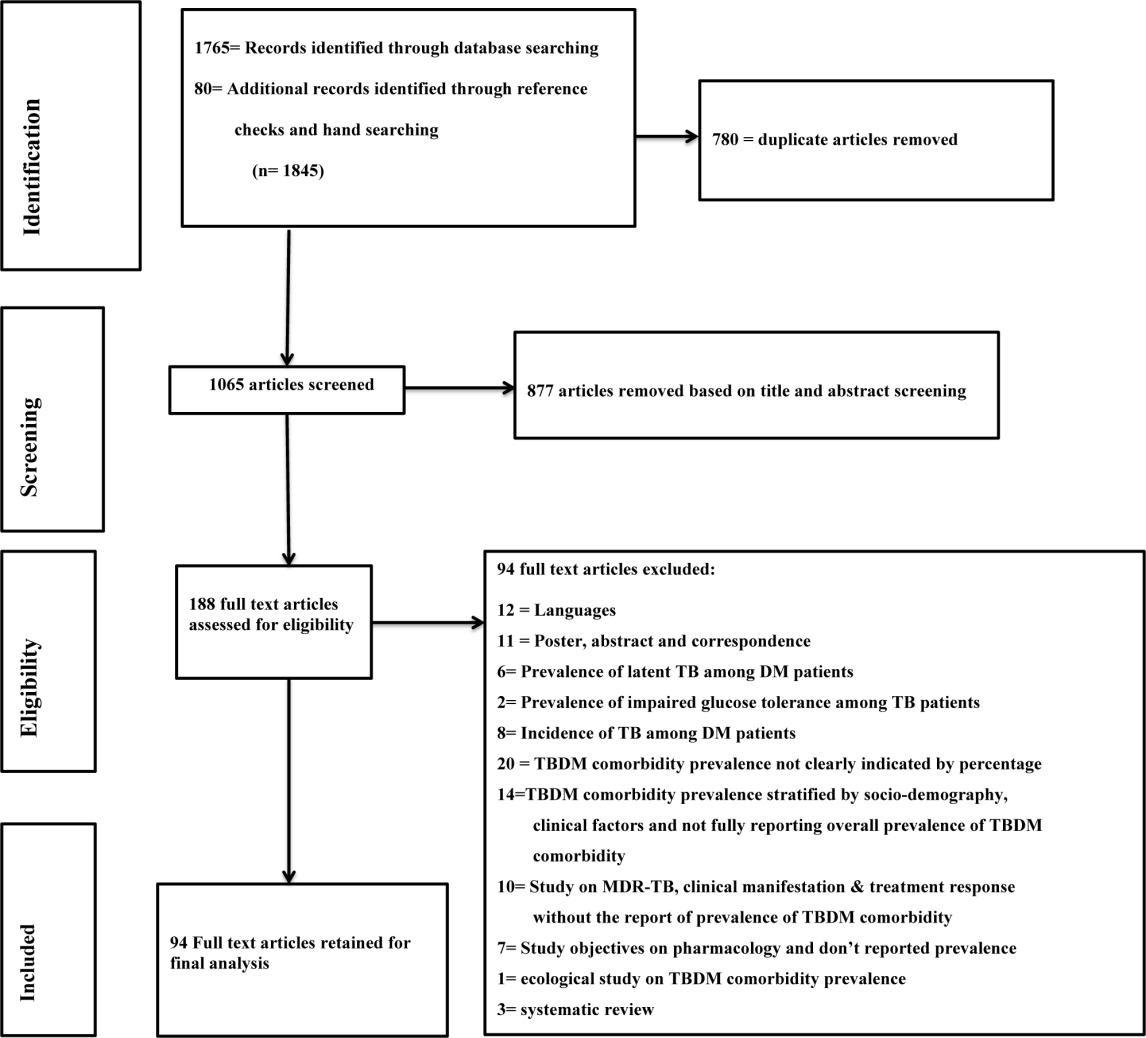
Flow diagram of searched articles. TB: tuberculosis, DM: diabetes mellitus, MDR: multi drug resistance.

A total of 1845 literatures were initially selected for screening. These included 1765 literatures identified from the electronic database search, 59 identified by hand search and 21 literatures identified by reference check **(Fig 1).** After removing 780 duplicate articles form the total 1845 literatures, 1065 articles remained for further screening. **Additional screening** by title and abstract resulted in the exclusion of 877 articles and we were left with 188 articles for further screening. We performed full text screening on 188 articles and found that 94 articles were eligible for final analysis [9–10, 14–105]. The criteria for exclusion of the different studies are listed in Fig 1.

The 94 studies selected for final analysis had applied different study designs. Majority 36 (38.3%) were cross-sectional studies, 11 (11.7%) were prospective cohort, 9 (9.6%) applied retrospective cohort study design, 21 (22.3%) were medical record reviews, four (4.3%) studies used prospective observational (not clearly specified) method, three (3.2%) used case-controls study design and 10 (10.6%) studies did not describe the type of study design used. The studies represented 33 countries globally and were divided into six regions. Of the total studies included in the analysis, 74 (78.7%) were published between 2011 and 2016, 12 (12.8%) studies were published from 2000 to 2010, and 8 (8.5%) studies were reported between 1957 to 1999 **(Table 3).**

**Table 3:** Profile of reviewed articles.

Region	First author and publication year	Country	Study period	Study design	Reference
Asia	India group et al. 2013	India	Jan-Sep/2012	Prospective observational	[9]
	China group et al. 2012	China	1 Sep 2011–31 March 2012	Prospective observational	[10]
	Achanta et al. 2013	Saluru-South India	Jan–Sep/2012	Cross-sectional	[14]
	Alavi et al. 2012	Iran	2008–2010	Medical record review	[15]
	Alisjahbana et al. 2006	Indonesia	2001–2005	Case-control	[16]
	Baghaei et al.2015	Tehran-Iran	May 2012-May 2013	Prospective cohort	[17]
	Balakrishnan et al.2012	Kerala-India	June-July/2011	Cross-sectional	[18]
	Dave et al. 2013	Gujarat -India	Jan-Sep/ 2012	Cross-sectional	[19]
	Jawad et al. 1995	Nazimabad-Pakistan	NR	NR	[20]
	Lin et al. 2015	Taiwan	Sep-Nov/2012	Cross-sectional	[21]
	Pandya et al. 1991	Riyadh	July 83-August 87	Medical record review	[22]
	Raghuraman et al. 2014	Puducherry	NR	Cross-sectional	[23]
	Rajapakshe et al. 2015	SriLanka	Jan 2013-Oct 2014	Cross-sectional	[24]
	Shidam et al. 2015	Pondicherry, India	Jan- Dec /2013	Cross-sectional	[25]
	Thapa et al. 2015	Nepal	15^th^ of Sep - 23rd of Nov/2013	Cross-sectional	[26]
	Usmani et al. 2014	Lahore-Pakistan	1^st^ July-3oth Sep /2011	Cross-sectional	[27]
	Viswanathan et al. 2012	India	Jan-March/2011	NR	[28]
	Nagar et al. 2015	India	Oct 2013-March 2014	Cross-sectional	[29]
	Wang et al. 2013	China	Sep 2010-Dec 2012	Prospective community based cohort	[30]
	Sarvamangala et al. 2014	India	Jan 2012-Aug 2012	Cross-sectional	[31]
	Deshmukh et al. 1984	India	NR	NR	[32]
	Chachra et al. 2014	Ghaziabad -India	NR	Cross-sectional	[33]
	Wang et al. 2000	Taiwan	1993–1996	Retrospective cohort	[34]
	Chaudhry et al. 2012	Filipino-Saudi-Arabia	Jan. 2003-June 2010	Retrospective/medical record	[35]
	Duangrithi et al. 2013	Thailand	April 2010 -July 2012	Prospective	[36]
	Jabbar et al.2006	Pakistian	Jan 1992-Dec 1996	Retrospective descriptive (Medical record review)	[37]
	Jali et al. 2013	India	Feb-Sep/2012	Cross-sectional	[38]
	Magee et al. 2015	Georgia- Tbilisi	Oct 2011-May 2014	Prospective cohort	[39]
	Mi et al. 2013	Guangzhou, China	1 Sep. 2011–30 June 2012	Cross-sectional and retrospective cohort study (medical record review)	[40]
	Mi et al. 2014	Bejing -China	1 Jan 2011–30 June 2012	Cross-sectional and retrospective record review	[41]
	Pablo-Villamor et al.2014	Philippines	July 2011-Nov.2012	Prospective observational cohort study	[42]
	Park et al.2012	Korea	Jan 2005-Dec 2009	Retrospective (medical record review)	[43]
	Roghieh et al. 2011	Iran	2004–2008	Retrospective cross-sectional (review of medical record)	[44]
	Mehta et al. 2015	India	2012–2013	Cross-sectional	[45]
	Shaikh et al. 2003	Saudi-Arabia	Jan1998-Dec1999	Retrospective (medical record)	[46]
	Siddiqui et al. 2009	Saudi-Arabia	Jan 2002-Dec 2007	Retrospective (medical record review)	[47]
	Sulaiman et al. 2013	Malaysia	Jan 2006-Dec 2007	Retrospective cohort	[48]
	Zhang et al. 2009	China	2008–2009	Retrospective	[49]
	Chen et al. 2014	China	Jan 2010-Dec 2011	Cross-sectional	[50]
	Jali et al. 2013	India	Feb 2012-Sep 2012	Prospective observational study	[51]
	Kumpatla et al. 2013	India	Mar-Dec/ 2012	Descriptive (review of record)	[52]
	Tripathy et al. 1984	India	1^st^ Jan. 1978- 31^st^ Dec.1982	Prospective study	[53]
	Wu et al. 2015	China	2007–2008	Retrospective population based study	[54]
	Naeem et al. 2016	Pakistan	Feb 2013-Dec 2014	Prospective observational	[55]
	Nair et al. 2013	Kerala-India	March-Sep/2012	Descriptive study	[56]
	Tahir et al.2014	Kohat-Pakistan	NR	Cross-sectional	[57]
	Jain et al. 2015	India	NR	Cross-sectional	[58]
	Amin et al. 2011	Pakistan	1^st^ Aug 2010-31st July 2011	NR	[59]
	Prakash et al.2013	India	1 March-30 Sep 2012	Descriptive study	[60]
	Qayyum et al.2004	Pakistan	Jan.2001 –Dec 2001	NR	[61]
	Sangral et al. 2012	Jammu-India	2009–2010	NR	[62]
	Alisjahbana et al. 2007	Indonesia	Oct 2000-Dec 2005	Prospective cohort	[63]
	Kermansaravi et al. 2014	Iran	April 2010-Dec 2011	Cross-sectional	[64]
	Padmalatha et al. 2014	India	May 2014-Oct 2014	Cross-sectional	[65]
	Kottarath et al. 2015	Kerala-India	Aug.2014-July 2015	Descriptive	[66]
	Rao et.al 2015	Hyderabad-India	June-July/ 2014	Cross-sectional	[67]
Africa	Ade et al. 2015	Cotonou-Benin	June-July/ 2014	Cross-sectional	[68]
	Amare et al. 2013	Ethiopia	Feb-April/ 2012	Cross-sectional	[69]
	Faurholt-Jepsen et al. 2011	Tanzania	April 2006-Jan 2009	Case control	[70]
	Haraldsdottir et al. 2015	Guinea-Bissau	July 2010-July 2011	NR	[71]
	Kibirige et al. 2013	Uganda	Sep 2011- Feb 2012	Cross-sectional	[72]
	Mtwangambate et al. 2014	Tanzania	Sep 2011-March 2012	Prospective cohort	[73]
	Ogbera et al. 2014	Lagos-Nigeria	Sep 2010 –March 2012	Cross-sectional	[74]
	Olayinka et al. 2013	Lagos-Nigeria	NR	Cross-sectional	[75]
	Workneh et al.2016	Ethiopia	Sep 2103 –Sep 2014	Cross-sectional	[76]
	Feleke et al. 1999	Ethiopia	Sep 1989–1996	Cross-sectional based on the retrospective analysis of data review record	[77]
	Swai et al. 1990	Tanzania	1 June 1981–31 May 1977	NR	[78]
	Webb et al. 2009	South -Africa	10 Sept 2006–31 Jan 2007	Cross-sectional	[79]
	Kirui et al. 2012	Kenya	Jan 2007-Feb 2011	Descriptive study from routine record data	[80]
	Tiroro et al. 2015	Ethiopia	Jan 2010-Jan 2014	Retrospective study (medical record)	[81]
	Ogbera et al. 2015	Lagos-Nigeria	March 2011-July 2012	Descriptive observational study	[82]
	Getachew et al. 2014	Ethiopia	Oct.2011-August 2012	Cross-sectional	[83]
	Damtew et al. 2014	Ethiopia	Feb.2014-May 2014	Cross-sectional	[84]
	Balad et al. 2006	Guinea	1 Feb 30 -June 2002	NR	[85]
	Rakotonirina et al. 2014	Antananarivo-Madagascar	July15,2013—Oct.30,2013	Descriptive	[86]
	Mugusi et al. 1990	Tanzania	NR	NR	[87]
Europe	Moreno-Mart´ınez et al. 2015	European city- Barcelona	1 Jan 2000–31 Dec 2013	Retrospective, population based cross-sectional	[88]
	Warwick et al. 1957	Britain	1 Jan 1940-Dec 31,1954	Medical record review	[89]
North America	Ponce-de-leon et al. 2004	Mexico	1995–2003	Population based cohort study	[90]
	Restrepo et al. 2007	Texas-Mexico	Mexico (1998–2003) / Texas (1996–2002)	Medical record review	[91]
	Restrepo et al. 2011	South-Texas & North -eastern Mexico	March 2006-Sep 2008	Cross-sectional	[92]
	Magee et al. 2014	Georgia-US	Jan 2009- Sep 2012	Retrospective cohort	[93]
	Suwanpimolkul et al.2014	USA-San Francisco	April 2005-March 2012	Retrospective	[94]
	Delgado-Sánchez et al. 2015	Mexico	2000–2012	TB registry review retrospective analysis	[95]
	Castellanos-Joya et al. 2014	Mexico	July 2012—April 2013	Prospective observational cohort	[96]
	Jiménez-Corona et al. 2013	Southern -Mexico	1995 to 2010	Prospective cohort	[97]
South America	Alladin et.al. 2011	Guyana	May-June/2006	Cross-sectional	[98]
	Magee et al. 2013	Peru	Jan.2005-May 2008	Medical record	[99]
	Reis-Santos et al. 2013	Brazil	2009	Disease notification information system	[100]
Oceania	Bridison et al. 2015	Australia	1995–2014	Retrospective	[101]
	Viney et al. 2015	Kiribati-Pacific Island	June 2010-March 2012	Case control (unmatched)	[102]
	Nasa et. al. 2014	Ebeye-Marshall Islands	July 2010-Dec 2012	Retrospective cohort	[103]
	Prasad et al. 2014	Fiji	2010–2012	Retrospective descriptive (TB register)	[104]
	Gounder et al. 2012	Fiji	Jan-March/2012	Cross-sectional medical record review	[105]

NR = Not reported, TB = tuberculosis.

### Risk of bias

The value of risk of bias ranged from 1 to 5 for 78 studies that determined prevalence of DM among TB patients. Based on this assessment, 23 (29.5%) studies were assessed to have low risk of bias, 49 (62.8%) studies had moderate risk of bias and 6 (7.7%) studies were evaluated as having high risk of bias. The risk of bias for 19 studies that analyzed prevalence of TB among DM patients ranged from 0 to 4. Accordingly, 3 (15.8%) studies were assessed to have low risk of bias, 8 (42.1%) studies were evaluated as having moderate risk of bias and 8 (42.1%) studies were assessed to have high risk of bias **(-S1 Table. Assessment of risk of bias of the studies).**

### Prevalence of DM among TB patients

**Fig 2:**
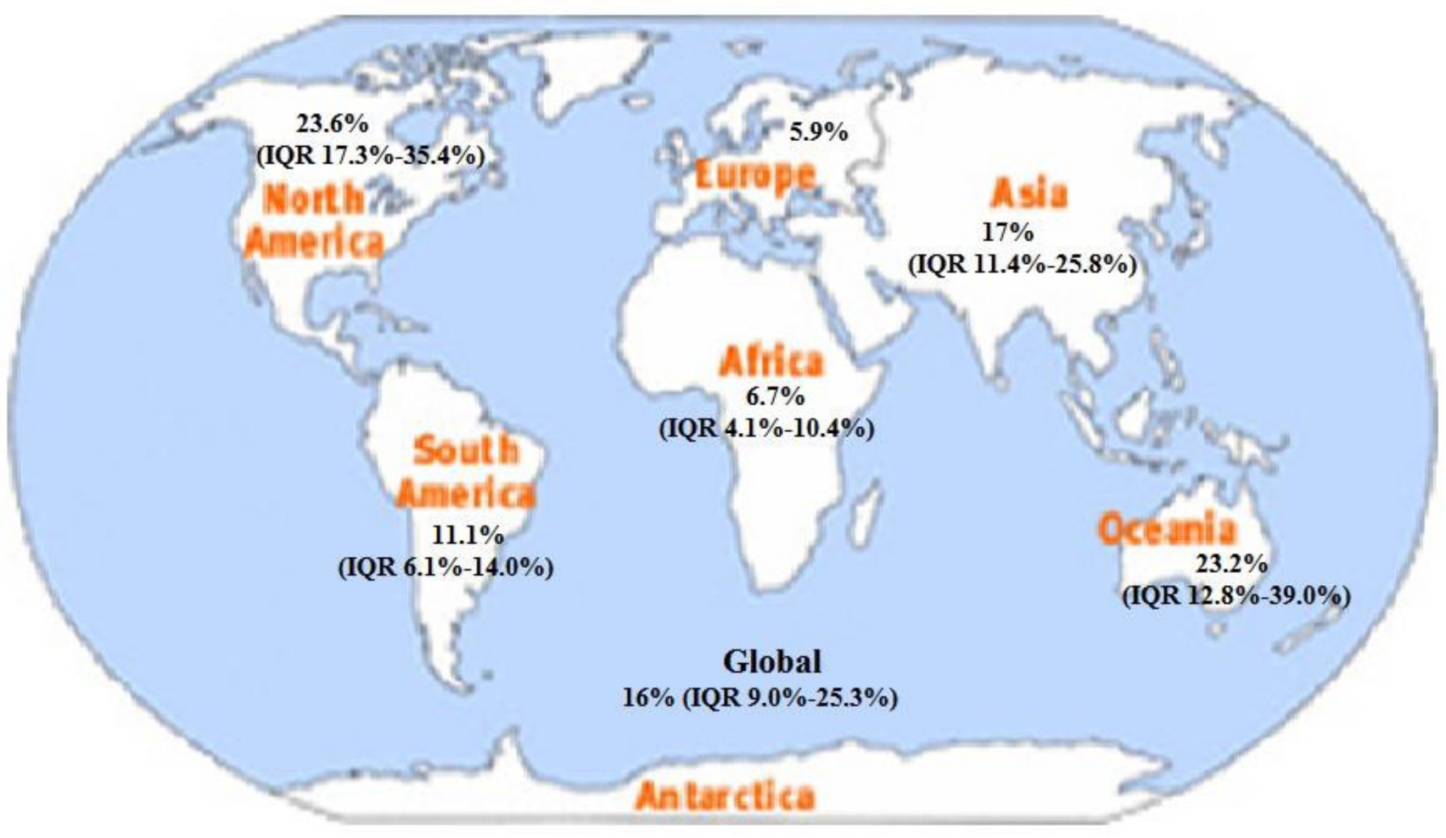
Map showing median prevalence of DM among TB patients by region. (NB: There is only one study reported in Europe). IQR: Interquartile range (Source of the map: https://www.flickr.com/photos/blatantworld/5052373414#. Accessed March 20/2017).

Out of the total 94 studies, 78 studies reported DM prevalence among TB patients. Except one study, all reported the total number of observed DM cases among enrolled TB patients. Accordingly, the prevalence of DM among TB patients ranged from 1.9% in Cotonou-Benin to 45% in Ebeye-Marshall Islands [68,103]. This amounted to an overall global median DM prevalence of 16% (IQR 9.0–25.3%). Among the 78 studies, 48 (61.5%) studies were conducted in countries of Asia and showed prevalence rates ranging from 5.1% in Saluru-South India to 44% in Kerala-India [14, 18]. The overall median prevalence of DM among TB patients in Asia was calculated to be 17% (IQR 11.4%-25.8%). Thirteen (16.7%) studies conducted in countries of Africa showed prevalence rates ranging from 1.9% in Cotonou-Benin to 16.7% in Tanzania [68, 70]. This resulted in an overall median prevalence of 6.7% (IQR 4.1%-10.4%) in the studied countries of Africa. Eight (10.3%) studies that were done in countries of North America showed a prevalence rates ranging from 11.4% in Georgia [93] to 39.0% in South Texas [92]. The median prevalence in North America was 23.6% (IQR 17.3%-35.4%). There were five (6.4%) studies from Oceania that showed prevalence rates ranging from 12% in Fiji [104] to 45% in Ebeye-Marshall Islands [103] and the overall median prevalence in this area was 23.2% (IQR 12.8%-39.0%). Three (3.8%) studies conducted in South America indicated prevalence rates ranging from 6.1% in Brazil to 14% in Guyana [98,101]. This amounted to an overall median prevalence of 11.1% (IQR 6.1%-14.0%). There was only one study from Europe that showed a prevalence rate of 5.9% **(Fig 2).**

Of the 78 studies included in this systematic review, only ten (12.8%) studies reported number needed to screen (NNS). The NNS indicates the number of TB patients that must be screened to get a single case of DM. The NNS ranged from four cases in Kerala India [18] to 56 in SriLanka [23]. Only 42 (53.8%) studies described the number of new DM patients obtained by screening TB patients. The number of new DM cases obtained after screening ranged from one case in Cotonou-Benin [68] to 402 cases in India [9] **(Table 4).**

**Table 4:** Prevalence of TBDM comorbidity.

Region	First author and publication year	Country	Prevalence of DM among TB patients						Prevalence of TB among DM patients				Reference
			Enrolled TB patients	Total DM case	Prevalence of DM (%)	Type of DM status and prevalence		NNS					
						New N (%)	Known N (%)		Enrolled DM patients	Total TB cases	Prevalence of DM (%)	NNS	
Asia	India group et al. 2013	India	8109	1084	13	402 (5)	682 (8)	NR					[9]
	China group et al. 2012	China	8886	1090	12.4	227 (2.9 )	863 (9.7 )	NR					[10]
	Achanta et al. 2013	Saluru-South India	374	19	5.1	12 (3.2)	7 (1.9)	31					[14]
	Alavi et al. 2012	Iran	148	36	24.3	NR	36 (24.3)	NR					[15]
	Alisjahbana et al. 2006	Indonesia	454	60	13.2	36 (60.0)	24 (40)	NR					[16]
			556 Non-TB control	18	3.2	12 (66.7)							
	Baghaei et al.2015	Tehran-Iran	293	101	34.5	45 (15.4)	56 (19.1)	5					[17]
	Balakrishnan et al.2012	Kerala-India	552	243	44	115 (21)	128 (23)	4					[18]
	Dave et al. 2013	Gujarat -India	553	36	6.5	22 (4)	14 (2.5)	25					[19]
	Jawad et al. 1995	Nazimabad-Pakistan	106	21	19.8	NR	NR	NR					[20]
	Lin et al. 2015	Taiwan							3,087	12	0.38	NR	[21]
	Pandya et al. 1991	Riyadh	1566	136	8.7	NR	NR	NR					[22]
	Raghuraman et al. 2014	Puducherry	217	63	29	18 (8.3)	45 (20.7)	NR					[23]
	Rajapakshe et al. 2015	SriLanka	112	10	9	2 (1.8)	8 (7.1)	56					[24]
	Shidam et al. 2015	Pondicherry, India	570	121	21.2 (95% CI 18.0– 24.7)	52 (43)	69 (57)	11					[25]
	Thapa et al. 2015	Nepal	407	37	9.1	9 (2.2)	28 (6.9)	NR					[26]
	Usmani et al. 2014	Lahore-Pakistan	158	41	25.9	9 (5.69)	32 (20.3)	NR					[27]
	Viswanathan et al. 2012	India	827	209	25.3	77 (9.3)	132 (15.96)	NR					[28]
	Nagar et al. 2015	India	220	34	15.4	9 (4.09)	25 (11.3)	NR					[29]
	Wang et al. 2013	China	6382	403	6.3	177 (43.9)	NR	NR					[30]
			6675 Non-TB controls	313	4.7	136 (43.5)	NR	NR					
	Sarvamangala et al. 2014	India	200	28	14	NR	18 (64.3)	NR					[31]
	Deshmukh et al. 1984	India	2434	138	5.6	78 (56.6)	60 (43.4)	NR					[32]
	Chachra et al. 2014	Ghaziabad -India	700	88	12.6	64 (72.7)	24 (27.3)	NR					[33]
	Wang et al. 2000	Taiwan	2841	480	16.9	NR	NR	NR					[34]
	Chaudhry et al. 2012	Filipino-Saudi -Arabia	1388	114	7.17	NR	NR	NR					[35]
	Duangrithi et al. 2013	Thailand	227	37	16.3	11 (29.7)	26 (70.3)	NR					[36]
	Jabbar et al.2006	Pakistan							1458	173	11.9	NR	[37]
									Non-DM 40,9000	691	1.7		
	Jali et al. 2013	India	307	109	35.5	NR	NR	NR					[38]
	Magee et al. 2015	Georgia- Tbilisi	318	37	11.6 (95% C.I. 8.4–15.5)	9 (24.3)	NR	NR					[39]
	Mi et al. 2013	Guangzhou, China	1589	189	12	NR	NR	NR					[40]
	Mi et al. 2014	Bejing -China	621	187	30	NR	NR	NR					[41]
	Pablo-Villamor et al.2014	Philippines	38	7	18.4 (95% C.I. 7.7–34.3)	NR	NR	NR					[42]
	Park et al.2012	Korea	492	124	25.2	NR	NR	NR					[43]
	Roghieh et al. 2011	Iran	200	80	40	NR	NR	NR					[44]
	Mehta et al. 2015	India	194	22	11.3	NR	NR	NR					[45]
	Shaikh et al. 2003	Saudi-Arabia	692	187	27	23 (12.3)	NR	NR					[46]
				505 controls without DM									
	Siddiqui et al. 2009	Saudi-Arabia	216	35	16	NR	NR	NR					[47]
	Sulaiman et al. 2013	Malaysia	1267	338	26.7	NR	NR	NR					[48]
	Zhang et al. 2009	China	2141	203	9.5	NR	NR	NR					[49]
	Chen et al. 2014	China	1126	182	16.2	18 (1.6)	164 (14.6)	NR					[50]
	Jali et al. 2013	India	307	109	35.5	49 (15.96 )	60 (19.54)	NR	4118	111	2.70	NR	[51]*
	Kumpatla et al. 2013	India							7083	50	0.7	NR	[52]
	Tripathy et al. 1984	India							219	9	4.1	NR	[53]
	Wu et al. 2015	China	201	40	19.90	NR	NR	NR					[54]
	Naeem et al. 2016	Pakistan	95	17	16.75	NR	NR	NR					[55]
	Nair et al. 2013	Kerala-India	920	298	32.4	63 (7)	235 (26)	NR					[56]
	Tahir et al.2014	Kohat-Pakistan	253	48	18.97	NR	NR	NR					[57]
	Jain et al. 2015	India	189	41	21.69	NR	NR	NR					[58]
	Amin et al. 2011	Pakistian							100	14	14	NR	[59]
	Prakash et al.2013	India	510	47	9.2	15 (2.9 )	32 (6.3)	16	1670	47	2.8	812	[60]*
	Qayyum et al.2004	Pakistan							95	9	9.5	NR	[61]
									96 Non-DM	2	2.08		
	Sangral et al. 2012	Jammu-India	280	23	8.2	NR	NR	NR					[62]
	Alisjahbana et al. 2007	Indonesia	634	94	14.8	57 (61.3)	NR	NR					[63]
	Kermansaravi et al. 2014	Iran							400	1	1	NR	[64]
	Padmalatha et al. 2014	India	252	77	30.60	60 (77.8)	17 (22.2)	NR					[65]
	Kottarath et al. 2015	Kerala -India	147	29	19.7	16 (55)	13 (45)	NR					[66]
	Rao et.al 2015	Hyderabad-India							96	10	10	NR	[67]
Africa	Ade et al. 2015	Cotonou-Benin	159	3	1.9	1 (0.63)	2 (1.26)	NR					[68]
	Amare et al. 2013	Ethiopia							225	14	6.2	NR	[69]
	Faurholt-Jepsen et al. 2011	Tanzania	803	NR	16.7 (95% C.I. 14.2–19.4)	NR	NR	NR					[70]
			350 Non-TB control	NR	9.4 (95% C.I. 6.6–13.0)								
	Haraldsdottir et al. 2015	Guinea-Bissau	107	3	2.8	NR	NR	NR					[71]
			531 Non- TB control	11	2.1								
	Kibirige et al. 2013	Uganda	260	22	8.5	NR	5 (1.9)	NR					[72]
	Mtwangambate et al. 2014	Tanzania							693	9	1.3	NR	[73]
	Ogbera et al. 2014	Lagos-Nigeria	3376	162	4.8	85 (52.5)	77 (47.5)	NR					[74]
	Olayinka et al. 2013	Lagos-Nigeria	351	20	5.7	10 (2.8)	NR	NR					[75]
	Workneh et al.2016	Ethiopia	1314	109	8.3	64 (4.9)	45 (3.4)	19.8					[76]
	Feleke et al. 1999	Ethiopia							1352	78	5.8	NR	[77]
	Swai et al. 1990	Tanzania							1250	70	5.6	NR	[78]
	Webb et al. 2009	South -Africa							258	9	3.48	NR	[79]
	Kirui et al. 2012	Kenya							1376	77	5.6	NR	[80]
	Tiroro et al. 2015	Ethiopia							681	26	3.8 (95% C.I. 2.5- 5.3)	NR	[81]
	Ogbera et al. 2015	Lagos-Nigeria	4000	480	12.3	310 (7.7)	170 (4.3)	NR					[82]
	Getachew et al. 2014	Ethiopia	199	17	8.5 (95% C.I. 4.6–12.5)	9 (53)	NR	NR					[83]
	Damtew et al. 2014	Ethiopia	120	19	15.8 (95% C.I. 9.20–22.45)	16 (84.2 )	3 (15.8 )	NR					[84]
	Balad et al. 2006	Guinea	388	13	3.35 (95% C.I. 1.35–5.35)	4 (31)	NR	NR					[85]
	Rakotonirina et al. 2014	Antananarivo-Madagascar	156	9	5.8 (95% C.I. 3.1–10.6)	NR	4 (2.6)	NR					[86]
	Mugusi et al. 1990	Tanzania	506	34	6.7	25 (4.9)	9 (1.8)	NR					[87]
Europe	Moreno-Mart´ınez et al. 2015	European city- Barcelona	5849	349	5.9	NR	NR	NR					[88]
	Warwick et al. 1957	Britain							1851	34	1.82	NR	[89]
North America	Ponce-de-leon et al. 2004	Mexico	525	185	35.2	NR	NR	NR					[90]
	Restrepo et al. 2007	Texas	1441	401	27.8	NR	401 (27.8)	NR					[91]
		Mexico	3411	607	17.8	NR	607 (17.8)						
	Restrepo et al. 2011	South-Texas	61	24	39.0	NR	NR	NR					[92]
		North -eastern Mexico	172	62	36.0	NR	NR	NR					
	Magee et al. 2014	Georgia-US	1325	151	11.4	NR	NR	NR					[93]
	Suwanpimolkul et al.2014	USA-San Francisco	791	126	15.9	NR	NR	NR					[94]
	Delgado-Sánchez et al. 2015	Mexico	181,378	34,988	19.29	NR	34,988 (19.29 )	NR					[95]
	Castellanos-Joya et al. 2014	Mexico	361	70	19.4	16 (22.9 )	NR	22	783	38	4.9	71	[96]*
	Jiménez-Corona et al. 2013	Southern -Mexico	1262	400	31.7	26 (2.1)	374 (29.6)	NR					[97]
South America	Alladin et.al. 2011	Guyana	100	14	14	2 (14.3)	12 (85.7)	NR					[98]
	Magee et al. 2013	Peru	1671	186	11.1	NR	NR	NR					[99]
	Reis-Santos et al. 2013	Brazil	29,275	1797	6.1 (95% C.I. 5.9–6.4)	NR	NR	NR					[100]
Oceania	Bridison et al. 2015	Australia	69	16	23.2	NR	NR	NR					[101]
	Viney et al. 2015	Kiribati-Pacific Island	275	101	37	47 (17.1)	54 (19.6)	5					[102]
			499 control	94	19	61 (12)	33 (7.0)	8					
	Nasa et. al. 2014	Ebeye-Marshall Islands	62	28	45	NR	NR	NR					[103]
	Prasad et al. 2014	Fiji	567	68	12	8 (11.8)	26 (38.2)	NR					[104]^θ^
	Gounder et al. 2012	Fiji	138	18	13	NR	18 (13)	NR					[105]

* = bidirectional screening studies results

^θ^ = the types of DM status information for 34 (50%) patients was not documented

DM = diabetes mellitus, TB = tuberculosis, NNS = number needed to screen, NR = not reported, C.I. = confidence interval.

### Prevalence of TB among DM patients

**Fig 3:**
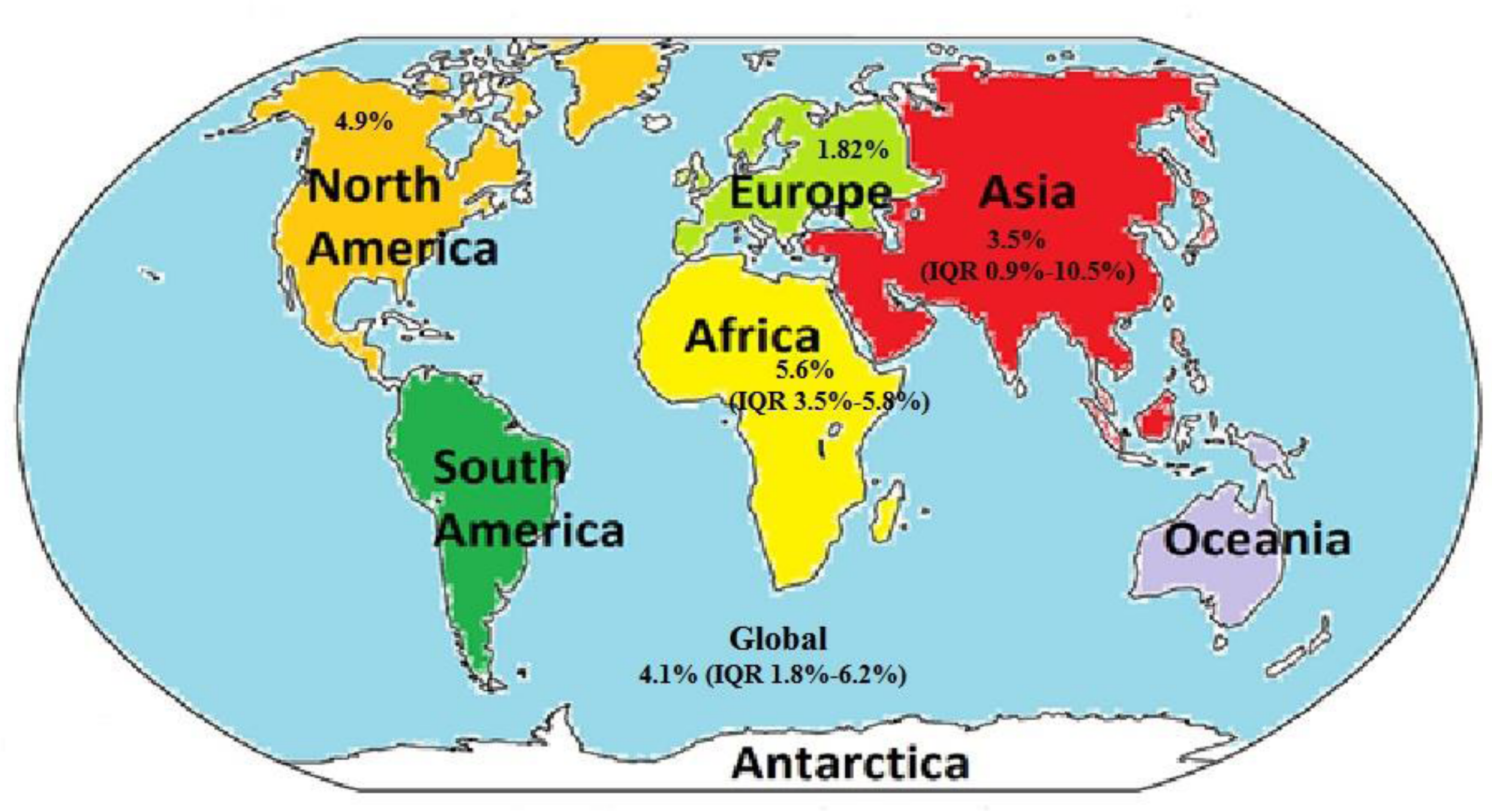
Map showing median prevalence of TB among DM patients by region. (NB: North America and Europe each reported only one study). IQR: Interquartile range (Source of the map: http://www.sawyoo.com/postpic/2015/02/what-are-the-7-seven-continents_118851.png. Accessed March 20/2017).

Out of the total 94 studies, 19 studies reported TB prevalence among DM patients. The studies were conducted in11 countries distributed in four geographic regions of the world. The prevalence of TB among DM patients ranged from 0.38% in Taiwan [21] to 14% in Pakistan [59], and the overall median prevalence was 4.1% (IQR 1.8%-6.2%). Among the 19 studies, ten (52.6%) were from four countries of the Asian Region and the prevalence ranged from 0.38% in Taiwan [21] to 14% in Pakistan [59]. This amounted to an overall median TB prevalence of 3.5% (IQR 0.9%-10.5%) among DM patients in the studied countries of Asian Region. Seven (36.8%) prevalence studies were conducted in four countries of the African Region, and the prevalence ranged from 1.3% in Tanzania [73] to 6.2% in Ethiopia [69]. The overall median TB prevalence among DM patients in the Africa studies was 5.6% (IQR 3.5%-5.8%). There was only one study in North America (Mexico) that showed a prevalence rate of 4.9% [96]. There was also one prevalence study from Europe that showed prevalence rate of 1.82% [89] **(Fig 3).** Only two study reported the NNS and NNS reported to screen DM patients to get one TB case ranges 71 DM patients in Mexico[96] to 812 in India **[60] (Table 4).**

### Risk factors for TBDM comorbidity

The risk/associated factors for TBDM comorbidity were heterogeneous. Both sexes, age, family history of DM, pulmonary form of TB (PTB) and positive sputum smear were the most frequently mentioned factors in the majority of the studies. The studies used different measures of association to analyze the factors. Out of the 94 reviewed articles, 11(11.7%) studies applied chi square test. Twenty two (23.4%) studies reported TBDM risk/associated factors using odds ratio, relative risk or hazard ratio. Conversely, 61 (64.9%) studies did not report either associated or risk factors for TBDM coexistence. This might be due to that most of the studies did not have adequate sample size and almost all studies mentioned neither in their objective nor in the limitation part about the risk factors of TBDM comorbidity. The following is a brief thematic description of the associated/ risk factors that were identified in the different studies.

#### 1. Socio-demographic and economic factors

A number of studies concluded that both males [18, 21, 94] and females [76, 91, 95] were at increased risk for TBDM comorbidity. Men were more likely to develop TBDM comorbidity compared to women [15]. Twenty two studies reported that older age increased the risk of TBDM comorbidity [14–15, 17–19, 23, 25–26, 28, 30, 48, 54, 56, 65–67, 76, 81, 86, 91, 96, 100]. Urban residence and having an education level beyond primary schooling were associated factors for TBDM comorbid conditions [31, 69]. Place of birth, ethnicity, high-income status and sedentary occupation were risk factors associated with TBDM comorbidity [21, 26, 28, 30, 48, 86, 91, 94].

#### 2. Behavioral factors

Illicit drug use, and sedentary lifestyle were reported as behavioral factors associated with TBDM comorbidity [15, 85]. Cigarette smoking [21] and being alcohol drinker [23] were identified as an increased risk factors for TBDM coexistence. Practicing frequent outdoor activity was reported as a low behavioral risk factor for TBDM comorbidity [30].

#### 3. Clinical factors

Both lower and higher body mass index (BMI), human immune–deficiency virus (HIV) coinfection, body weight loss and hypertension were reported as associated factors for TBDM comorbidity [15–16, 21, 26, 58, 65, 85]. Both lower and higher BMI were also reported as an increased risk factor for TBDM comorbidity [28, 81]. Pre-existing and long duration of DM [69, 81], poor glycemic control at the time of TB diagnosis [79], patients with liver cirrhosis [21] and history of high blood pressure [26] were identified as an increased risk factor for the development of TBDM comorbidity. DM with both positive [15] and negative [70] HIV status- were documented as associated and increased risk factor for TBDM comorbid condition. HIV coinfection and malnutrition were also reported as low risk factor for TBDM comorbidity [72, 95]. HIV coinfection with injection drug use (IDU) or without IDU was reported as low risk factor for TBDM coexistence [88]. TBDM comorbid patients were more likely to be PTB case, smear- positive, to have anti-TB drug resistance, to have cavitary lesions on chest x-ray, and to have high alanine transaminase (ALT) level [10, 25, 28, 30, 48, 54, 56, 88–89, 72, 76, 84, 88, 95, 100]. On the contrary, being an extra pulmonary TB (EPTB) case was reported as a low risk factor for TBDM comorbidity [100].

#### 4. History of DM, TB illness & TB treatment

Having family history of DM, history of TB illness and treatment, experiencing more side effect of anti-TB treatment, type of TB treatment category, treatment for previous TB episode and extension of anti-TB treatment durations were reported as increased risk factor for TBDM comorbidity [23, 25–26, 28, 30, 54, 65, 69, 76, 88,95]. Receiving TB treatment after abandonment was also identified as low risk factor for TBDM comorbidity [100].

#### 5. Other factors

Contact with TB patient in the family was reported as associated/ increased risk factor for TBDM comorbidity [16, 69, 79]. Being imprisoned was associated with TBDM comorbidity [15, 98]. TBDM comorbid patients may require hospitalization [88]. TBDM patients were more likely to die from TBDM comorbidity [100]. Being kept in certain institutions (prisons shelter, orphanage and psychiatric hospital) were documented as low risk factor for TBDM comorbidity [100] **(Table 5).**

**Table 5:** Thematic analysis of risk/associated factors for TBDM comorbidity.

Risk factor	Associated factors	Risk factors	
		Increased Risk	Low risk
**Socio-demography and economic factors**			
**Sex**			
Male	[15]	[18,21,94]	
Female		[76,91,95]	
**Age**			
Old age	[14–15,17,19,65,67,98]	[18,23,25–26,28,30,48,54,56,76,81,88,91,100]	
Urban residence	[31]	[69]	
Education beyond primary schooling	[31]		
Place of birth (Spanish born, Chinese, Philippines)		[48,88,94]	
Ethnicity (Hispanic)		[91]	
High-income status		[26,30]	
Sedentary occupation		[28]	
Family size	[67]		
**Behavioral factors**			
Illicit drug use	[15]		
Sedentary lifestyle	[85]		
Smoking		[21,26]	
Current alcohol drinker		[23]	
Frequent outdoor activity			[30]
**Clinical factor**			
Body weight loss		[21]	
BMI [17.7 kg/m^2^ (range11.2–31.4), (<18.6kg/m^2^), (18.5–22.9 kg/m2), and (≥18.5 kg/m^2^)]	[16]	[28,81]	
Overweight or obese	[58,65,85]		
DM		[81]	
Long duration of DM		[69,81]	
Poor glycemic control per unit increase in glycated hemoglobin(HbA1c)		[79]	
DM in HIV negative status		[70]	
HIV coinfection	[15]		[72]
HIV with injection drug use			[88]
HIV without injection drug use			[88]
Malnutrition			[95]
Liver cirrhosis		[21]	
Hypertension	[65]	[26]	
PTB		[28,48,56,76]	
EPTB			[100]
Drug resistance (in patient with antimicrobial susceptibility test)		[95]	
Positive sputum smear		[25,30,54,84,100]	
Cavitary on chest X-ray		[30,54,88]	
Raised serum ALT concentration		[72]	
Treatment for diabetes	[67]		
**History of DM, TB illness & treatment**			
Family history of DM	[85, 98]	[23,25–26,28,30,76]	
History of TB illness		[69]	
TB treatment category	[65]		
Experiencing more side effect of anti-TB treatment		[88]	
To receive TB treatment after abandonment^*^			[100]
Treatment for a previous TB episode (in patient without antimicrobial susceptibility test)		[95]	
Extension of anti-TB treatment duration		[54]	
**Other factors**			
Contact with TB patient in the family	[16]	[69,79]	
Imprisonment	[15,98]		
Hospitalization		[88]	
Institutionalization^θ^			[100]
Outcome of TB: death		[100]	

* ^=^ subjects that discontinued previous TB treatment and returned to treatment

^θ =^ being in prison shelter, orphanage and psychiatric hospital

TB = tuberculosis, DM = diabetes mellitus, BMI = body mass index, HIV = human immune-deficiency virus, PTB = pulmonary tuberculosis, EPTB = extra pulmonary tuberculosis, ALT = alanine transaminase, HbA1c = Hemoglobin A1c, kg = kilogram, m^2^ = meter square.

## Discussion

This systematic review revealed that the global burden of TBDM comorbidity is high, and is fueled by heterogeneous risk/associated factors. The observed global TBDM comorbidity prevalence in the current systematic review is higher compared to the findings of the previous systematic review conducted in 2010 [11]. This might be related to the increasing number of studies addressing TBDM comorbidity in the last six years. A total of 74 studies have been published since 2011 which showed a threefold increase compared to the number of similar studies conducted before 2010. Contrary to the previous systematic review [12], where studies from the Africa Regions were not reported, our systematic review showed an increasing number of studies reporting high prevalence of DM among TB patients in some countries of the African Region.

The number of new DM patients identified by screening TB patients varied in the different studies. This variation might be due to differences in the screening methods used and variations in the prevalence of DM in the general population of the respective countries. However, the large proportion of newly identified DM patients suggests the identification of previously undiagnosed DM patients and highlights that screening TB patients for DM in the TB clinic is an important public health intervention [102].

The observed prevalence of TB among DM patients in this systematic review is low compared to the previous systematic review findings [11]. This might be related to the small number of similar studies conducted, the low sensitivity of diagnostic methods used to detect TB cases and the magnitude of TB prevalence in the studied countries. In addition, the language restriction criteria that we used may have resulted in underreporting bias. Hence, we must be cautious in the interpretation of this finding. The prevalence of TB among DM patients in the studied countries of Asia and the African Regions were high compared to findings of other regions. This may be linked to the fact that countries in these continents are experiencing the fastest increase in DM prevalence along with the high burden of TB and HIV [27, 72].

We analyzed socio-demographic, behavioral, clinical and other factors associated with TBDM comorbidity. Male sex was identified as a risk/associated factor for TBDM comorbidity. Men usually practice smoking cigarettes and alcohol drinking which can predispose them to both diseases conditions [84]. Similarly, being women was found to be risk factor for TBDM comorbidity. The reason may be linked to poor health service utilization, care taking role of women for the sick, and influence of estrogen on cytokine production during TB infection that increases the vulnerability of women to TB and consequently to DM [76]. Old age was reported as associated/risk factor for TBDM comorbidity. The reason may be related to decrease in immune status in older age individuals that make them more susceptible to develop both TB and DM [48, 76, 81]. High-income status was also identified as risk factor for the two comorbid condition [26, 30]. Patients with high-income may spend much time in sedentary lifestyle activities than their counter parts and have better access for diagnostic and medical facilities [26]. Urban residence was reported as associated/risk factor for the development of TBDM comorbid condition [31, 69]. This might be due to the overcrowded living conditions, less physical activity and consumption of a high calorie rich diet among residents in urban areas [69]. In addition, urban residents have better access for the diagnosis of TB and DM.

Behavioral attributes such as tobacco smoking and alcohol drinking are associated with TBDM comorbidity [21, 23, 26]. Cigarette smoking results in inflammation and oxidative stress in body cells and increases the risk of developing DM [26**]**. In contrast, frequent outdoor activity was identified as protective factor for TBDM comorbidity [30]. This might be linked to the fact that increased physical activity results in increased peripheral insulin sensitivity which leads to more glucose uptake by body muscles [26].

Our systematic review identified various clinical factors associated with TBDM comorbidity. Patients BMI status was identified as increased as well as low risk factor for TBDM comorbid conditions. Previous studies showed that overweight and obesity were risk factors for DM but were protective against TB disease. However, weight loss due to poorly controlled DM and metabolic decomposition takes away this protection and becomes risk factor for TB [30, 81]. Existing DM was the other risk factor for TBDM coexistence. Long term DM is usually associated with uncontrolled DM and can impair the innate and adaptive immune response necessary to counteract the proliferation of TB [28, 69, 81]. Poor glycemic control and high blood pressure were reported as risk factors for TB among DM patients [79]. In resource poor settings, early diagnosis and adequate glycemic control is difficult and poor glycemic control may predispose DM patients to TB disease. In addition, hyperglycemia may provide a conducive environment for bacterial growth and increased virulence of various organisms [69–70, 79, 81]. The increased risk factor for TBDM related to high blood pressure may be linked to the fact that persons with DM were more likely to develop high blood pressure [26].

There is contradictory finding regarding the association of HIV with TBDM comorbidity [15, 70, 72, 88]. This might be linked to use of taking cotrimoxazole prophylaxis among HIV positive patients. Cotrimoxazole has been found to cause hypoglycemic effects in some patients [72]. The risk factor related to HIV infection could also be related to use of certain antiretroviral drugs that may predispose HIV infected patients to DM [106]. Having family history of DM was also identified as associated/risk factors for TBDM comorbidity. Family history of DM is a known risk factor for DM [3].

Contact with known TB patients was considered as risk factor for the development of TB among DM patients [16, 69, 79]. Frequent contact could lead to transmission of TB [69]. Patients with history of imprisonment were more likely to be exposed to TBDM comorbid conditions [15, 98].This might indicate that the acquisition of both diseases during imprisonment period is very high [98] and might be related to overcrowded and stressful living conditions. It was also reported that TBDM comorbid patients usually become hospitalized [88]. DM patient more likely require hospitalization due to glycemic imbalance as a result of infection that may require taking insulin [88].

This systematic review has strengths and weaknesses. The comprehensive search strategy applied using multiple electronic databases and the inclusion of a large number of studies covering almost all geographic regions of the world are strengths of the study. Potential limitation of the study could be the exclusion of studies written in other languages except English. However, since our inclusion criteria was very broad and accommodated majority of the studies that assessed the magnitude and associated/risk factors of TBDM comorbidity, the effect of excluding non-English written articles in the generalizability of the study findings would be minimal. We could not be able to report age of study participants due to lack of uniformity in the way it was reported in the reviewed articles. We recommend future studies to address this important variable. One may question why we used prevalence rate to report the findings since all studies reviewed were not cross-sectional studies. However, majority of the articles included in this systematic review reported their findings as prevalence of either TB among DM or DM among TB patients. Some reported as the number of DM or TB patients obtained from screening TB or DM patients. The studies were observational studies and used cross-sectional and descriptive study designs. We thus have used prevalence rate as our effort was to relate it with what the reviewed articles reported. We did not exclude studies based on the level of risk of bias assessment as our main objective was to understand the global picture of the prevalence and associated/risk factors of TBDM comorbidity in a more comprehensive manner. We believe that this may not significantly affect the generalizability of the study as majority of the studies were evaluated as having low-moderate risk of bias. We did not perform metanalysis because of methodological variations observed in the different studies included in our systematic review. The studies varied by type of study design used, methods of DM and TB screening, timing of DM screening and number of enrolled patients.

## Conclusion

This systematic review revealed that there is a high burden of DM among TB patients at global level. The highest prevalence of DM among TB patients is observed in the studied countries of Asia, North America and Oceania. On the contrary, the prevalence of TB among DM patients is low globally, but relatively higher in the studied countries of Asia and the African continents. Factors associated with TBDM comorbidity included sex, older age, urban residence, illicit drug use, alcoholism, cigarette smoking, sedentary lifestyle, obesity, HIV coinfection, hypertension, long duration of pre-existing DM, poor glycemic control, being a PTB patient, and family history of DM.

The implementation of the WHO recommended TBDM integrated services is important to address the impact of TBDM comorbidity [6]. However, as implementing such a strategy is resource intensive, countries may benefit by first assessing the magnitude and risk/associated factors of TBDM comorbidity before making decisions to undertake such a big initiative.

## Supporting information

S1 Prisma Checklist This is prisima checklist for the prevalence and associated factors of tuberculosis and diabetes mellitus comorbidity: a systematic review. (DOCX)

S1 Table Assessment of risk of bias of the studies. (DOCX)
