# Supplementary material for: Prevalence and associated factors of tuberculosis and diabetes mellitus comorbidity: A systematic review
Source: PLoS One. 2017 Apr 21;12(4):e0175925. doi: 10.1371/journal.pone.0175925 (PMC5400500; doi:10.1371/journal.pone.0175925)
Supplement: S1 Table — (DOCX) [file pone.0175925.s002.docx]

**(S1- Table . Assessment of risk of bias of the studies)**

| Region | First author and publication year | Prevalence of DM | | | | | | Prevalence of TB | | | | | Reference |
| --- | --- | --- | --- | --- | --- | --- | --- | --- | --- | --- | --- | --- | --- |
|  |  | Risk of bias domain | | | | | | Risk of bias domain | | | | |  |
|  |  | Study design | Sampling methods | Methods of DM screening | Time of DM screening | Sample size detrmination | Total risk | Study design | Sampling methods | TB screening methods | Sample size detrmination | Total risk |  |
| Asia | India group et al. 2013 | 0 | 1 | 0 | 0 | 1 | 2 |  |  |  |  |  | [ 9] |
|  | China group et al. 2012 | 0 | 1 | 0 | 0 | 1 | 2 |  |  |  |  |  | [10] |
|  | Achanta et al. 2013 | 0 | 1 | 0 | 1 | 1 | 3 |  |  |  |  |  | [14] |
|  | Alavi et al. 2012 | 1 | 1 | 0 | 1 | 1 | 4 |  |  |  |  |  | [15] |
|  | Alisjahbana et al. 2006 | 0 | 1 | 0 | 0 | 1 | 2 |  |  |  |  |  | [16] |
|  | Baghaei et al.2015 | 0 | 1 | 0 | 0 | 1 | 2 |  |  |  |  |  | [17] |
|  | Balakrishnan et al.2012 | 0 | 1 | 0 | 0 | 0 | 1 |  |  |  |  |  | [18] |
|  | Dave et al. 2013 | 0 | 1 | 0 | 0 | 1 | 2 |  |  |  |  |  | [19] |
|  | Jawad et al. 1995 | 1 | 1 | 0 | 1 | 1 | 4 |  |  |  |  |  | [20] |
|  | Lin et al. 2015 |  |  |  |  |  |  | 0 | 1 | 0 | 1 | 2 | [21] |
|  | Pandya et al. 1991 | 1 | 1 | 0 | 1 | 1 | 4 |  |  |  |  |  | [22] |
|  | Raghuraman et al. 2014 | 0 | 1 | 0 | 1 | 1 | 3 |  |  |  |  |  | [23] |
|  | Rajapakshe et al. 2015 | 0 | 1 | 0 | 1 | 1 | 3 |  |  |  |  |  | [24] |
|  | Shidam et al. 2015 | 0 | 1 | 0 | 0 | 0 | 1 |  |  |  |  |  | [25] |
|  | Thapa et al. 2015 | 0 | 1 | 0 | 1 | 0 | 2 |  |  |  |  |  | [26] |
|  | Usmani et al. 2014 | 0 | 0 | 0 | 1 | 0 | 1 |  |  |  |  |  | [27] |
|  | Viswanathan et al. 2012 | 1 | 0 | 0 | 1 | 0 | 2 |  |  |  |  |  | [28] |
|  | Nagar et al. 2015 | 0 | 1 | 0 | 1 | 1 | 3 |  |  |  |  |  | [29] |
|  | Wang et al. 2013 | 0 | 0 | 0 | 1 | 0 | 1 |  |  |  |  |  | [30] |
|  | Sarvamangala et al. 2014 | 0 | 1 | 0 | 0 | 1 | 2 |  |  |  |  |  | [31] |
|  | Deshmukh et al. 1984 | 1 | 1 | 0 | 1 | 1 | 4 |  |  |  |  |  | [32] |
|  | Chachra et al. 2014 | 0 | 1 | 0 | 1 | 1 | 3 |  |  |  |  |  | [33] |
|  | Wang et al. 2000 | 1 | 1 | 0 | 1 | 1 | 4 |  |  |  |  |  | [34] |
|  | Chaudhry et al. 2012 | 1 | 1 | 0 | 1 | 0 | 3 |  |  |  |  |  | [35] |
|  | Duangrithi et al. 2013 | 0 | 1 | 0 | 1 | 0 | 2 |  |  |  |  |  | [36] |
|  | Jabbar et al.2006 |  |  |  |  |  |  | 1 | 1 | 0 | 0 | 2 | [37] |
|  | Jali et al. 2013 | 0 | 1 | 0 | 1 | 1 | 3 |  |  |  |  |  | [38] |
|  | Magee et al. 2015 | 0 | 1 | 0 | 1 | 1 | 3 |  |  |  |  |  | [39] |
|  | Mi et al. 2013 | 1 | 1 | 0 | 1 | 1 | 4 |  |  |  |  |  | [40] |
|  | Mi et al. 2014 | 1 | 1 | 0 | 1 | 1 | 4 |  |  |  |  |  | [41] |
|  | Pablo-Villamor et al.2014 | 0 | 1 | 0 | 1 | 1 | 3 |  |  |  |  |  | [42] |
|  | Park et al.2012 | 1 | 1 | 0 | 1 | 1 | 4 |  |  |  |  |  | [43] |
|  | Roghieh et al. 2011 | 1 | 1 | 0 | 1 | 1 | 4 |  |  |  |  |  | [44] |
|  | Mehta et al. 2015 | 0 | 1 | 1 | 1 | 1 | 4 |  |  |  |  |  | [45] |
|  | Shaikh et al. 2003 | 1 | 1 | 0 | 1 | 1 | 4 |  |  |  |  |  | [46] |
|  | Siddiqui et al. 2009 | 1 | 1 | 0 | 1 | 1 | 4 |  |  |  |  |  | [47] |
|  | Sulaiman et al. 2013 | 1 | 1 | 1 | 1 | 1 | 5 |  |  |  |  |  | [48] |
|  | Zhang et al. 2009 | 1 | 1 | 1 | 1 | 1 | 5 |  |  |  |  |  | [49] |
|  | Chen et al. 2014 | 0 | 1 | 0 | 1 | 1 | 3 |  |  |  |  |  | [50] |
|  | Jali et al. 2013 | 0 | 1 | 0 | 1 | 1 | 3 | 0 | 1 | 0 | 1 | 2 | [51]* |
|  | Kumpatla et al. 2013 |  |  |  |  |  |  | 1 | 1 | 0 | 1 | 3 | [52] |
|  | Tripathy et al. 1984 |  |  |  |  |  |  | 0 | 1 | 0 | 1 | 2 | [53] |
|  | Wu et al. 2015 | 1 | 1 | 1 | 0 | 1 | 4 |  |  |  |  |  | [54] |
|  | Naeem et al. 2016 | 0 | 1 | 0 | 1 | 1 | 3 |  |  |  |  |  | [55] |
|  | Nair et al. 2013 | 0 | 1 | 0 | 1 | 1 | 3 |  |  |  |  |  | [56] |
|  | Tahir et al.2014 | 0 | 0 | 0 | 1 | 1 | 2 |  |  |  |  |  | [57] |
|  | Jain et al. 2015 | 0 | 1 | 0 | 1 | 1 | 3 |  |  |  |  |  | [58] |
|  | Amin et al. 2011 |  |  |  |  |  |  | 1 | 1 | 0 | 1 | 3 | [59] |
|  | Prakash et al.2013 | 0 | 1 | 0 | 1 | 1 | 3 | 0 | 1 | 0 | 1 | 2 | [60]* |
|  | Qayyum et al.2004 |  |  |  |  |  |  | 1 | 1 | 0 | 1 | 3 | [61] |
|  | Sangral et al. 2012 | 1 | 1 | 0 | 1 | 1 | 4 |  |  |  |  |  | [62] |
|  | Alisjahbana et al. 2007 | 0 | 1 | 0 | 0 | 1 | 2 |  |  |  |  |  | [63] |
|  | Kermansaravi et al. 2014 |  |  |  |  |  |  | 0 | 1 | 0 | 1 | 2 | [64] |
|  | Padmalatha et al. 2014 | 0 | 1 | 0 | 1 | 1 | 3 |  |  |  |  |  | [65] |
|  | Kottarath et al. 2015 | 0 | 1 | 0 | 1 | 1 | 3 |  |  |  |  |  | [66] |
|  | Rao et.al 2015 |  |  |  |  |  |  | 1 | 0 | 1 | 0 | 2 | [67] |
| Africa | Ade et al. 2015 | 0 | 1 | 0 | 1 | 1 | 3 |  |  |  |  |  | [68] |
|  | Amare et al. 2013 |  |  |  |  |  |  | 0 | 1 | 0 | 0 | 1 | [69] |
|  | Faurholt-Jepsen et al. 2011 | 0 | 1 | 0 | 1 | 1 | 3 |  |  |  |  |  | [70] |
|  | Haraldsdottir et al. 2015 | 1 | 1 | 0 | 1 | 0 | 3 |  |  |  |  |  | [71] |
|  | Kibirige et al. 2013 | 0 | 1 | 0 | 0 | 0 | 1 |  |  |  |  |  | [72] |
|  | Mtwangambate et al. 2014 |  |  |  |  |  |  | 0 | 0 | 0 | 0 | 0 | [73] |
|  | Ogbera et al. 2014 | 0 | 1 | 0 | 1 | 1 | 3 |  |  |  |  |  | [74] |
|  | Olayinka et al. 2013 | 0 | 1 | 0 | 1 | 0 | 2 |  |  |  |  |  | [75] |
|  | Workneh et al.2016 | 0 | 1 | 0 | 0 | 0 | 1 |  |  |  |  |  | [76] |
|  | Feleke et al. 1999 |  |  |  |  |  |  | 1 | 1 | 0 | 1 | 3 | [77] |
|  | Swai et al. 1990 |  |  |  |  |  |  | 1 | 1 | 0 | 1 | 3 | [78] |
|  | Webb et al. 2009 |  |  |  |  |  |  | 0 | 1 | 0 | 0 | 1 | [79] |
|  | Kirui et al. 2012 |  |  |  |  |  |  | 1 | 1 | 1 | 1 | 4 | [80] |
|  | Tiroro et al. 2015 |  |  |  |  |  |  | 1 | 1 | 0 | 1 | 3 | [81] |
|  | Ogbera et al. 2015 | 0 | 1 | 0 | 1 | 1 | 3 |  |  |  |  |  | [82] |
|  | Getachew et al. 2014 | 0 | 1 | 0 | 1 | 0 | 2 |  |  |  |  |  | [83] |
|  | Damtew et al. 2014 | 0 | 1 | 0 | 1 | 0 | 2 |  |  |  |  |  | [84] |
|  | Balad et al. 2006 | 0 | 0 | 0 | 1 | 0 | 1 |  |  |  |  |  | [85] |
|  | Rakotonirina et al. 2014 | 0 | 1 | 0 | 1 | 1 | 3 |  |  |  |  |  | [86] |
|  | Mugusi et al. 1990 | 1 | 1 | 0 | 1 | 1 | 4 |  |  |  |  |  | [87] |
| Europe | Moreno-Mart´ınez et al. 2015 | 1 | 1 | 0 | 1 | 1 | 4 |  |  |  |  |  | [88] |
|  | Warwick et al. 1957 |  |  |  |  |  |  | 1 | 1 | 0 | 1 | 3 | [89] |
| North America | Ponce-de-leon et al. 2004 | 0 | 1 | 0 | 1 | 1 | 3 |  |  |  |  |  | [90] |
|  | Restrepo et al. 2007 | 1 | 1 | 1 | 1 | 1 | 5 |  |  |  |  |  | [91] |
|  | Restrepo et al. 2011 | 0 | 1 | 0 | 1 | 1 | 3 |  |  |  |  |  | [92] |
|  | Magee et al. 2014 | 1 | 1 | 1 | 0 | 1 | 4 |  |  |  |  |  | [93] |
|  | Suwanpimolkul et al.2014 | 1 | 1 | 1 | 1 | 1 | 5 |  |  |  |  |  | [94] |
|  | Delgado-Sánchez et al. 2015 | 1 | 1 | 1 | 1 | 1 | 5 |  |  |  |  |  | [95] |
|  | Castellanos-Joya et al. 2014 | 0 | 1 | 0 | 0 | 1 | 3 | 0 | 1 | 0 | 1 | 2 | [96]* |
|  | Jiménez-Corona et al. 2013 | 0 | 1 | 0 | 1 | 1 | 3 |  |  |  |  |  | [97] |
| South America | Alladin et.al. 2011 | 0 | 1 | 0 | 0 | 1 | 2 |  |  |  |  |  | [98] |
|  | Magee et al. 2013 | 1 | 1 | 0 | 1 | 1 | 4 |  |  |  |  |  | [99] |
|  | Reis-Santos et al. 2013 | 1 | 1 | 1 | 1 | 1 | 5 |  |  |  |  |  | [100] |
| Oceania | Bridison et al. 2015 | 1 | 1 | 0 | 1 | 1 | 4 |  |  |  |  |  | [101] |
|  | Viney et al. 2015 | 0 | 1 | 0 | 1 | 0 | 2 |  |  |  |  |  | [102] |
|  | Nasa et. al. 2014 | 1 | 1 | 0 | 1 | 1 | 4 |  |  |  |  |  | [103] |
|  | Prasad et al. 2014 | 1 | 1 | 0 | 1 | 1 | 4 |  |  |  |  |  | [104] |
|  | Gounder et al. 2012 | 1 | 1 | 0 | 1 | 1 | 4 |  |  |  |  |  | [105] |

0: low risk, 1: high risk *: bidirectional studies DM: diabetes mellitus, TB: tuberculosis
